# Leveraging unified multi-view hypergraph learning for neurodevelopmental disorders diagnosis

**DOI:** 10.3389/fmed.2025.1654199

**Published:** 2025-07-23

**Authors:** Xiangmin Han, Junchang Li

**Affiliations:** ^1^School of Software, Tsinghua University, Beijing, China; ^2^Shenzhen Clinical Research Center for Mental Disorders, Shenzhen Kangning Hospital and Shenzhen Mental Health Center, Shenzhen, China

**Keywords:** neurodevelopmental disorders, hypergraph learning, high-order correlation, brain disease, knowledge and data dual-driven

## Abstract

**Objective:**

Accurate diagnosis of neurodevelopmental disorders relies on understanding the complex interactions and high-order relationships between brain regions. This work aims to model the subtle, disease-specific high-order relationships among brain regions that have been overlooked in existing works.

**Method:**

This paper proposes a Unified Multi-View Hypergraph Learning framework that combines knowledge-driven and data-driven strategies for a more precise and comprehensive representation of the adolescent brain network. The knowledge-driven branch leverages prior knowledge of functional brain subnetworks to guide feature learning and uncover structured, high-order functional associations. Meanwhile, the data-driven branch consists of two complementary modules: at the global level, a nearest-neighbor-based strategy captures large-scale associations involving overlapping brain regions; at the local level, a granularity-adaptive approach identifies finer, region-specific high-order relationships, allowing for a more nuanced understanding of brain network interactions.

**Results:**

Experimental results on the ABIDE and ADHD datasets demonstrate that our method outperforms existing methods in diagnostic accuracy and robustness. Additionally, visualizing the high-order associations learned from both branches reveals new insights into the pathogenic mechanisms of these disorders.

**Conclusion:**

The proposed method combines knowledge-driven and data-driven strategies for high-order brain network modeling, advancing the understanding of brain networks in neurodevelopmental diseases.

## 1 Introduction

The human brain is a highly intricate network, where cognitive functions arise from the dynamic interactions between multiple brain regions (1). Functional magnetic resonance imaging (fMRI) enables researchers to observe these interactions in detail (2), representing them as functional brain networks.

Recent advances in brain network analysis have introduced graph-based models models (3–5), which represent brain regions as nodes and pairwise interactions as edges. While this graph-based approach provides a clearer structural representation of brain connectivity, it only captures low-order pairwise correlations between regions. Such a model is insufficient for understanding the intricate, higher-order interactions that are critical for cognitive processes and disease mechanisms. Specifically, these methods fail to model interactions involving three or more regions, which are often crucial for understanding the brain's complex behavior and abnormal network states in neurodevelopmental disorders.

To address this limitation, hypergraph-based methods have been proposed (6, 7). Unlike traditional graphs, which connect two nodes through edges, hypergraphs use hyperedges to directly connect multiple nodes. This enables the representation of high-order interactions (8), allowing for a richer and more detailed understanding of how groups of brain regions collaborate. However, existing hypergraph methods often rely on data-driven techniques, such as *k*-nearest neighbors (*k*NN) (8, 9), which result in fixed hyperedge sizes and predefined sets of nodes within each hyperedge. While these methods capture higher-order patterns, they often remain coarse, failing to reveal subtle and disease-specific high-order relationships, and may not fully reflect the complexity of brain connectivity.

To overcome these limitations, we propose a Unified Multi-View Hypergraph Learning (UMHL) framework. Our approach integrates both knowledge-driven and data-driven strategies within a dual-branch framework. The knowledge-driven branch utilizes prior knowledge of eight known functional brain subnetworks to guide the generation of high-order associations, ensuring that the learned features are biologically relevant. The data-driven branch consists of two modules: a global-level module that employs a nearest-neighbor-based strategy to capture large-scale associations involving overlapping brain regions, and a local-level module that adapts to finer, region-specific high-order relationships without overlap. These two branches are integrated through soft voting, allowing us to leverage the strengths of both approaches for more accurate brain disease diagnosis. The main contributions of this work are as follows:

1) We propose a Unified Multi-View Hypergraph Learning (UMHL) framework, combining knowledge- and data-driven strategies for capturing comprehensive high-order brain connectivity and disease-specific associations.
2) We propose a flexible global-local data-driven hypergraph construction method, which adaptively adjusts the granularity of interactions and captures more biologically meaningful high-order patterns compared to fixed-size hyperedge-based models.
3) We validate our framework through extensive experiments on the ABIDE and ADHD datasets, revealing novel, disease-specific high-order interaction patterns that provide insights into abnormal brain function and connectivity.

## 2 Related work

### 2.1 Graph neural networks

A graph structure is a mathematical model composed of nodes edges connecting these nodes (10), used to represent objects and their relationships. Graph neural networks (GNNs) leverage neural network techniques applied to the nodes and edges of the graph to effectively learn and extract features of individual nodes, edges, and the entire graph. This enables GNN and its variants variants (11–13) to achieve strong performance in tasks node classification, graph classification, and link prediction.

GNN-based methods treat brain regions as nodes and their connections as edges. Mapping fMRI data into a graph structure allows researchers to capture functional associations between brain regions. For example, Li et al. (4) introduced BrainGNN, which uses ROI-aware graph convolutional layers and ROI-selection pooling layers to identify key brain regions related to autism disease. Wang et al. (14) proposed adaptive multi-channel graph convolutional networks, which focus on integrating node features and topological structures to improve the performance of semi-supervised node classification tasks. These methods extend the traditional graph convolutional network paradigm and offer valuable tools for efficient modeling and analysis of brain network data.

### 2.2 Hypergraph neural networks

To address the limitations of traditional graph representations, researchers introduced hypergraphs. A hypergraph uses hyperedges that can connect any number of nodes, allowing for the natural modeling of high-order interactions among multiple brain regions (15). Hypergraph neural networks (HGNNs) can capture more complex patterns by aggregating and updating features from hyperedges and their associated nodes nodes (8, 16). This approach supports a more detailed analysis of high-order networks in the brain.

Hypergraphs and HGNNs have also been introduced to the analysis of brain networks (6, 7). For example, Greenspan et al. (17) introduced the BrainHGNN model, which uses ROI identity encoding and random-walk strategies to build a hypergraph. A self-learned weighted hypergraph convolution updates node and hyperedge features, revealing AD-related propagation patterns. Ji et al. (18) proposed a hypergraph attention network to model functional brain networks with *k*NN and *k*-means, by using node and hyperedge attention layers. However, the current hypergraph structure modeling method often relies on fixed data-driven strategies, resulting in fixed sets of nodes and edges. This rigid approach does not fully reflect the complex and multi-scale high-order interactions in real brain networks and ignores valuable domain knowledge. Combining domain knowledge with data-driven strategies to adaptively model high-order relationships from a global to a local scale remains a key challenge in brain network analysis.

## 3 Materials and methods

### 3.1 Overview

**Figure 1:**
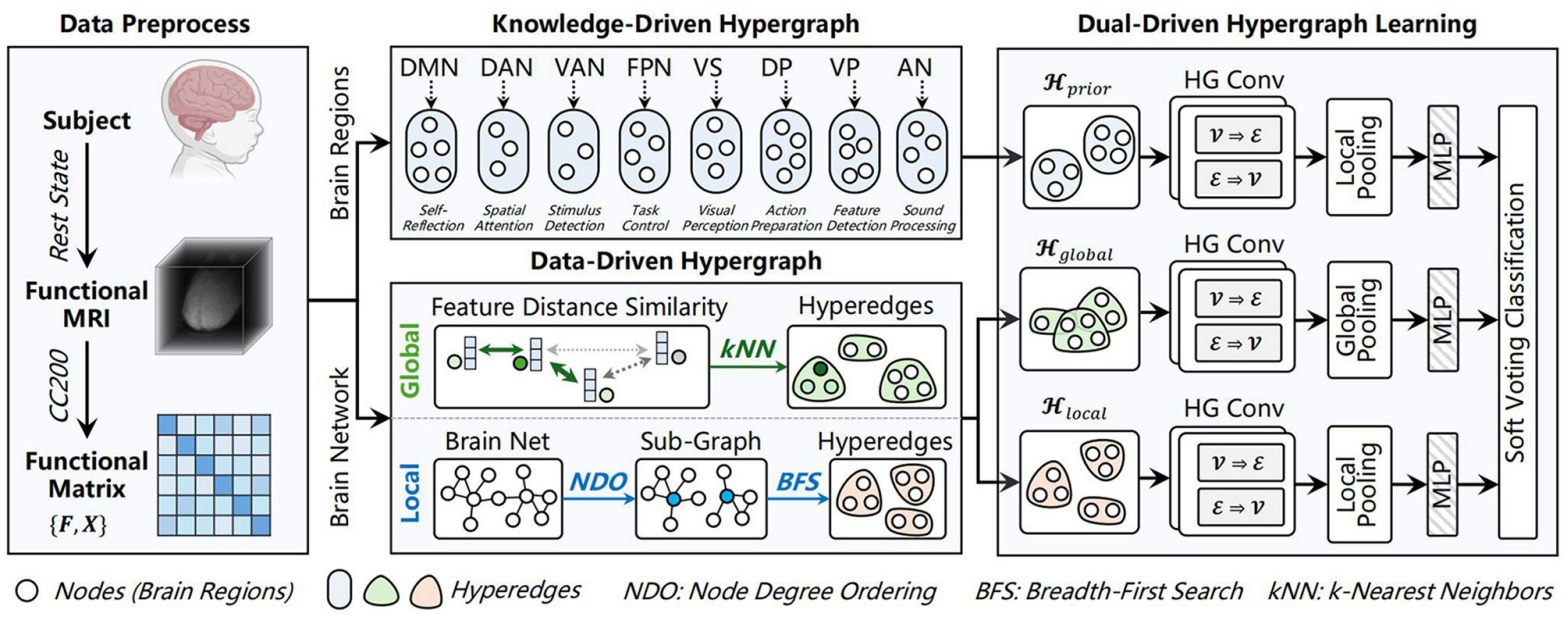
The proposed UMHL framework. UMHL consists of knowledge-driven and data-driven hypergraphs, both branches are combined through soft voting for classification. Created in BioRender. Han, X. (2025) https://BioRender.com/5z3tl1v.

As shown in Figure 1, the proposed UMHL framework has three key modules. In the knowledge-driven branch, a hypergraph is constructed using prior knowledge of eight functional brain subnetworks subnetworks (19) to capture high-order associations within each region. In the data-driven branch, global-local hypergraph modeling method that adapts the granularity of interactions. Both branches are combined through soft voting for brain disease classification.

### 3.2 Preliminaries of hypergraph

Consider a hypergraph H=(V,E), where V is the set of vertices and E is the set of hyperedges. The incidence matrix can be defined as $\text{H}\in {\mathbb{R}}^{\left|V|\times |E\right|}$, whose elements are determined as follows:


(1) $$H(v,e)=\{\begin{array}{ll} w_{e}(v), & \text{if }v\in e\\ 0 & \text{otherwise}, \end{array}$$

where *w*_*e*_(*v*) denotes the weight of vertex *v* in the hyperedge *e*. Unlike the adjacency matrix of a traditional graph, which is square, the incidence matrix of a hypergraph is not necessarily square. When |V|≠|E|, **H** forms a rectangular matrix. For the same dataset with fixed nodes, this characteristic enables the direct concatenation of different types of hyperedges, thereby creating fused hyperedges and a fused hypergraph structure (20).

### 3.3 Knowledge-driven hypergraph modeling

Initially, preprocessed fMRI data comprising blood oxygenation level-dependent (BOLD) signals are registered to a standardized brain template (21). Subsequently, Pearson's correlation coefficients are computed to derive the functional connectivity (FC) matrix representing pairwise associations between regions of interest (ROIs). This FC matrix serves as input to both computational branches.

Within the knowledge-driven hypergraph neural network (HGNN) branch, we integrate the derived FC features with a priori knowledge pertaining to eight pivotal functional brain regions. This integration facilitates the construction of a biologically grounded hypergraph structure, which explicitly models higher-order functional associations among these specialized subnetworks. Previous studies (19) have established that distinct brain regions subserve specialized cognitive and neural functions through collaborative interactions. By embedding these well-established neurobiological insights into the feature extraction framework, our model enhances the capacity to identify complex connectivity patterns and detect disease-associated alterations specific to individual subnetworks.

Formally, given the FC matrix **F**∈ℝ^*N*×*N*^, where *N* represents the number of ROIs, i.e., brain regions. Each ROI is treated as a node in the hypergraph, and its feature vector is defined as the row vector of **F** corresponding to that node. For a node *v*_*i*_ (corresponding to the *i*-th ROI), its feature vector is ${\text{x}}_{i}={\left[{\text{F}}_{i1},{\text{F}}_{i2},\ldots ,{\text{F}}_{iN}\right]}^{⊤}\in {\mathbb{R}}^{N}$, where **F**_*ij*_ denotes the functional connectivity strength between ROI *v*_*i*_ and ROI *v*_*j*_. Based on the prior knowledge of eight functional brain subnetworks, all *N* ROIs are partitioned into eight mutually exclusive functional brain subnetworks. Each subset is treated as a hyperedge *e*_*m*_, connecting all ROIs within that brain subnetwork. Thus, the hypergraph is constructed as:


(2) $$\begin{array}{l} H_{prior}=\left(V,E_{prior}\right),\;E_{prior}=\left\{e_{1},e_{2},\ldots ,e_{8}\right\}. \end{array}$$

### 3.4 Data-driven hypergraph construction

We introduce two modeling strategies to enrich multi-scale feature representations, i.e., the granularity-adaptive local modeling and the *k*NN-based global modeling. By leveraging the inherent characteristics of the data, the former constructs non-overlapping, fine-grained local topology hyperedges, and the latter constructs overlapping, global semantic hyperedges.

#### 3.4.1 Granularity-adaptive local modeling

The granularity-adaptive local modeling approach adopts a degree-correlated granularity partitioning strategy and implements recursive processing in a progressive manner. Leveraging the initial topological connections derived from the FC matrix, this method decomposes the brain network into mutually independent and non-overlapping fine-grained subnetworks. The extent of granularity decomposition is determined by topological connection constraints, ensuring that the resulting fine-grained subnetworks maintain maximal topological connectivity.

For the initial FC matrix **F**∈ℝ^*N*×*N*^, we used an threshold *t* to generates the corresponding adjacency matrix **A**∈ℝ^*N*×*N*^. Then, the degree of each node $D\left(v_{i}\right)$ is calculated and sorted in descending order. We follow Yu and Cheng (22) to selecte the top $S=\sqrt{N}$ nodes as the set of center nodes $V_{center}=\left\{v_{i}^{c}|i=1,...,S\right\}$. After selecting the center nodes, each non-center node $v\in V\backslash V_{center}$ is assigned to the subnetwork of the center node *p*_*i*_∈*P* with the shortest path distance. Once the assignment is complete, each subnetwork $S_{i}$ is defined as $G_{i}=\left(V_{i},E_{i}\right)$ containing the center node $v_{i}^{c}$ and all nodes assigned to it.

Next, a multi-source breadth-first search (BFS) algorithm is applied to reassign the nodes into two sub-subnetworks. Specifically, each initial $G_{i}$ is further refined to capture more detailed local topological structures. Specifically, the two nodes with the highest degrees *u* and *v* in $G_{i}$ are selected as the new center nodes for the sub-subnetwork, with the constraint as:


(3) $$\begin{array}{l} \left\{u,v\right\}=\arg\underset{x,y\in V_{i},x\neq y}{\max}\left(D\left(x\right),D\left(y\right)\right),\;\mathrm{Hop}\left(u,v\right)>2. \end{array}$$

where Hop(*u, v*) denotes the hop path between node *u* and *v*.

Inspired by Kim et al. (23), we define the average degree as the measure of topological connection strength by $\frac{2|E_{G}|}{\left|V_{G}\right|}$, where |·| denotes the number of elements in a set, $E_{G}$ and $V_{G}$ denotes the corresponding node set and edge set, respectively. If the total topology strength of the two sub-subnetworks $G_{l}$ and $G_{r}$ exceeds that of the original subnetwork $G_{i}$, the partitioning process continues recursively. Otherwise, the partitioning process stops. Additionally, if the number of nodes in a sub-subnetwork is 2 or if the partitioning depth reaches the predefined maximum depth *D*_max_, the recursion also terminates. Since we utilize the CC200 (21) brain template in this work, the *D*_max_ is set to 4. In this way, for each individual, a personalized set of fine-grained subnetworks can be adaptively generated based on the current individual's FC matric **F**, and these subnetworks are defined as hyperedges $E_{local}$ of a hypergraph $H_{local}=\left(V,E_{local}\right)$, with each hyperedge containing all nodes within the corresponding subnetwork. Notably, our method incorporates strict connection strength thresholds and recursion depth controls to prevent excessive fragmentation of the brain network. It does not blindly pursue smaller subnetwork sizes but rather balances the capture of fine-grained features with the integrity of the overall functional network, ensuring that the biological significance of key large-scale connections is not excessively diluted or fragmented. Furthermore, the termination conditions for recursive subdivision are designed to account for the connection density and biological meaningfulness of subnetworks, ensuring that the generated subnetworks retain effective interpretability.

The proposed granularity-adaptive local modeling method offers significant advantages by enabling flexible granularity partitioning that adapts to the complexity and heterogeneity of individual brain networks. This ensures that fine-grained subnetworks retain strong internal connectivity. Moreover, the recursive refinement process captures more elaborate local structures, thereby enhancing the model's capability to effectively represent functional connectivity patterns.

#### 3.4.2 *k*NN-based global modeling

Through the *k*NN, we are able to model globally interconnected complex structures, enabling the extraction of high-order global semantic associations from the FC matrix. Since both the knowledge-based hypergraph modeling and the granularity-adaptive local modeling generate non-overlapping hyperedges, they facilitate efficient message-passing within local subnetworks. However, to prevent message propagation within the high-order brain network from local optima, we also generate global semantic hyperedges based on the *k*NN algorithm.

Specifically, we define the similarity between each pair of ROIs based on the node features in the FC matrix **F**. The Euclidean distance is used to measure the similarity between nodes. For any two nodes *v*_*i*_ and *v*_*j*_, their similarity is given by ||**x**_*i*_−**x**_*j*_||_2_. Subsequently, for each node *v*_*i*_, the *k* nearest neighbors are selected to form a hyperedge that includes *v*_*i*_ and its *k* neighboring nodes. Specifically, the global hypergraph $H_{global}=\left(V,E_{global}\right)$ can be obtained.

It is important to emphasize that our *k*NN hypergraph modeling is not based on the spatial adjacency of brain regions, but rather on the feature similarity of functional connectivity to establish connections between regions. This means that even if brain regions are not spatially adjacent, they can be included in the same hyperedge if they exhibit significant similarity in the feature space of functional connectivity, thereby effectively capturing pathological patterns involving functionally correlated but spatially non-adjacent regions.

### 3.5 Dual-driven hypergraph learning

To summarize, we construct three types of hypergraph structures for each individual: a knowledge-driven hypergraph $H_{prior}=\left(V,E_{prior}\right)$, a granularity-adaptive local hypergraph $H_{local}=\left(V,E_{local}\right)$, and a *k*NN based global semantic hypergraph $H_{global}=\left(V,E_{global}\right)$. Since all three hypergraphs are built upon the same individual, they share the same node set V, i.e., brain regions).

For a hypergraph H=(V,E), where $H\in \left\{H_{prior},H_{local},H_{global}\right\}$, it can be denoted as a combination of a feature matrix and an incidence matrix, i.e., (**X**^(0)^, **H**). The hypergraph convolution process can be formally expressed as Gao et al. (8):

(4) $$\begin{array}{l} {\text{X}}^{\left(l+1\right)}=\delta \left(\text{H}\left(\mathrm{Mask}\left({\text{H}}^{⊤}{\text{X}}^{\left(l\right)}\Theta^{\left(l\right)}\right)\right)\right), \end{array}$$

where δ(·) denotes the nonlinear activation function, Mask(·) represents the hyperedge feature masking, and Θ^(*l*)^ is the learnable parameters. After several layers of hypergraph convolution, the learned node features **X**^(*L*+1)^ is obtained.

Next, we perform feature embedding on the three sets of node features. Since ${\text{X}}_{prior}^{\left(L+1\right)}$ and ${\text{X}}_{local}^{\left(L+1\right)}$ consist of mutually exclusive functional brain regions and fine-grained subnetworks respectively, a local pooling strategy is applied to these two representations. Specifically, for each subnetwork, the node feature vectors are mean pooled to generate the feature representation of the subnetwork, and the feature representations of all subnetworks are then concatenated to form the final feature representations **f**_*prior*_ and **f**_*local*_. For ${\text{X}}_{global}^{\left(L+1\right)}$, a global pooling is applied to obtain **f**_*global*_, where all node features are pooled using both max pooling and mean pooling, and the results are concatenated.

Finally, **f**_*prior*_, **f**_*local*_, and **f**_*global*_ are separately passed through multi-layer perceptrons (MLPs) and Softmax layers to generate logits for classification. The final classification decision is obtained through soft voting, where the logits are summed and a final softmax operation is applied:

(5) $$\begin{array}{l} {\text{Logits}}_{final}=\text{Softmax}\left({\text{Logits}}_{prior}+{\text{Logits}}_{local}+{\text{Logits}}_{global}\right). \end{array}$$

In summary, the knowledge-driven hypergraph focuses on capturing higher-order relationships within the predefined functional brain networks. In contrast, the data-driven hypergraphs (global and local) capture semantic relevance at the global scale and fine-grained topological structures at the local scale of the brain network, respectively. Consequently, the feature representations from these three distinct perspectives are inherently complementary rather than redundant. During the feature fusion stage, the use of soft voting further mitigates detrimental interference between branches, effectively ensuring model performance and robustness.

## 4 Results and discussion

### 4.1 Datasets and preprocessing

We used the Autism Brain Imaging Data Exchange (ABIDE) dataset (24) and the ADHD-200 dataset (25) in this work. The ABIDE dataset consists of 403 autism spectrum disorder (ASD) patients and 468 normal controls (NC). The ADHD-200 dataset consists of 362 ADHD patients and 585 NCs. Each subject contains at least one rs-fMRI scan. All the rs-fMRI are processed via the Data Processing Assistant for Resting-State fMRI (DPARSF) Tool with standard steps^1^. Subsequently, each subject's brain was parcellated into 200 regions of interest (ROIs) based on the CC200 atlas (21).

### 4.2 Experimental setup

All experiments were performed on an NVIDIA RTX 3090 GPU using PyTorch. The Adam optimizer was employed with a learning rate of 0.0005 and a weight decay of 0.00001. For hypergraph construction, the default parameter *k* of the global branch was set to 5. The model comprises two hypergraph convolutional layers and was trained for 100 epochs with early stopping.

### 4.3 Comparison and evaluation

The proposed method was evaluated to CNN-based method [BrainNetCNN (26)], GNN-based methods [BrainGNN (4), MVS-GCN (27), PLSNet (28), and BrainIB (5)], HGNN-based methods [HGNN (29) and HH-GF (30)].

The 5-fold cross-validation was used for all methods. The following metrics were selected for evaluation: accuracy, sensitivity, specificity, F1 score, and AUC. The final results are presented as the mean and standard error obtained during the 5-fold cross-validation process.

### 4.4 Results

The experimental results on the ABIDE dataset are shown in Table 1, which demonstrates that our proposed UMHL method outperforms existing graph-based and hypergraph-based methods. The main limitation of graph-based methods lies in their ability to capture only pairwise low-order correlations, which are insufficient to represent the complex multi-region interactions and higher-order correlations in brain networks. While hypergraph-based methods attempt to address this limitation by connecting multiple nodes with hyperedges, their reliance on data-driven strategies often fails to precisely capture the fine-grained, disease-specific higher-order relationships between brain regions. Our method combines both knowledge-driven and data-driven strategies, utilizing prior knowledge to guide the construction of hypergraphs while flexibly modeling both local and global relationships. As shown in Table 2, on the ADHD dataset, the results reveal that all methods exhibit higher specificity than sensitivity, which reflects the inherent characteristics and imbalance of the dataset. Despite this, our UMHL method shows remarkable performance, indicating its strong robustness and flexibility in handling imbalanced datasets.

**Table 1:** Experimental results of all compared methods on the ABIDE dataset.

**Method**	**ACC**	**SEN**	**SPE**	**F1**	**AUC**
BrainNetCNN	0.6473 ± 0.032	0.6278 ± 0.060	0.6627 ± 0.030	0.6454 ± 0.027	0.6686 ± 0.024
BrainGNN	0.6608 ± 0.026	0.6007 ± 0.021	0.6924 ± 0.039	0.6324 ± 0.024	0.6499 ± 0.035
HGNN	0.6595 ± 0.021	0.6286 ± 0.043	0.6787 ± 0.042	0.6803 ± 0.029	0.6615 ± 0.018
MVS-GCN	0.6813 ± 0.026	0.6922 ± 0.052	0.6716 ± 0.047	0.6771 ± 0.035	0.6804 ± 0.029
PLSNet	0.6860 ± 0.019	0.6846 ± 0.254	0.6889 ± 0.306	0.6860 ± 0.018	0.6996 ± 0.005
BrainIB	0.6921 ± 0.086	0.6532 ± 0.057	0.7294 ± 0.053	0.6791 ± 0.022	0.6902 ± 0.033
HH-GF	0.7005 ± 0.035	0.6958 ± 0.055	0.7154 ± 0.063	0.7012 ± 0.033	0.7126 ± 0.055
UMHL	0.7172 ± 0.015	0.7096 ± 0.014	0.7249 ± 0.020	0.7171 ± 0.015	0.7504 ± 0.015

**Table 2:** Experimental results of all compared methods on The ADHD-200 dataset.

**Method**	**ACC**	**SEN**	**SPE**	**F1**	**AUC**
BrainNetCNN	0.5548 ± 0.037	0.4864 ± 0.070	0.6024 ± 0.087	0.5562 ± 0.040	0.5721 ± 0.023
BrainGNN	0.6003 ± 0.016	0.4613 ± 0.053	0.6976 ± 0.033	0.5536 ± 0.050	0.5891 ± 0.044
HGNN	0.6167 ± 0.049	0.4876 ± 0.073	0.6958 ± 0.064	0.5745 ± 0.083	0.6008 ± 0.038
MVS-GCN	0.6313 ± 0.020	0.5351 ± 0.047	0.7017 ± 0.042	0.6343 ± 0.020	0.6176 ± 0.037
PLSNet	0.6215 ± 0.020	0.5248 ± 0.023	0.6843 ± 0.023	0.6215 ± 0.014	0.5972 ± 0.021
BrainIB	0.6358 ± 0.027	0.5275 ± 0.029	0.6952 ± 0.044	0.6158 ± 0.017	0.6039 ± 0.034
HH-GF	0.6547 ± 0.027	0.5324 ± 0.059	0.7331 ± 0.061	0.6332 ± 0.045	0.6287 ± 0.025
UMHL	0.6688 ± 0.017	0.5588 ± 0.020	0.7428 ± 0.028	0.6688 ± 0.017	0.6346 ± 0.021

Furthermore, the key hyperparameter *k* can highly affect the performance. Specifically, setting *k* too large increases the number of neighbor nodes connected to each brain region, potentially leading to an overly complex hypergraph structure that incorporates numerous irrelevant or weakly correlated regions. This redundancy could dilute genuinely pathological signals and reduce model precision. Conversely, setting *k* too small results in overly sparse connections between brain region nodes, potentially failing to adequately capture important disease-specific inter-regional interaction patterns, leading to insufficient sensitivity to key pathological features. Therefore, the *k* value needs to be judiciously selected within a moderate range based on specific data characteristics to achieve precise and effective feature capture.

**Figure 2:**
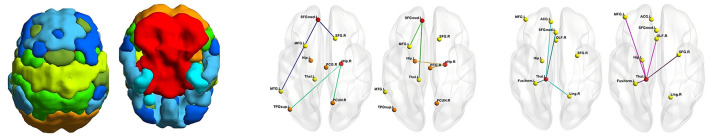
Significant hyperedge generated by different hypergraph modeling approaches. Left: prior hypergraph, middle: *k*NN hypergraph, right: granularity hypergraph.

Table 3 shows the ablation study results. The prior knowledge-driven model does not capture the complex inter-region relationships, leading to lower performance. The global interaction model improves the results by modeling large-scale associations, but it still lacks the fine-grained details provided by local interactions. When both local and global models are combined, the performance significantly improves, demonstrating that detailed, region-specific interactions are essential for accurate brain disease diagnosis. The full UMHL model leverages the strengths of each component, showing that combining prior knowledge, global, and local interactions provides a more robust and effective approach. As shown in Figure 2, we have visualized the significant hyperedges of different hypergraph modeling methods, where the hyperedges of the eight brain subnetworks are distinguished by color. Additionally, the stark contrast in significance between the two data-driven modeling approaches further underscores the importance of incorporating both global and local information to achieve a more comprehensive understanding.

**Table 3:** Ablation studies on ABIDE and ADHD-200 datasets.

**Datasets**	**Hypergraph**	**ACC**	**SEN**	**SPE**	**F1**	**AUC**
ABIDE	prior	0.6200 ± 0.023	0.6236 ± 0.028	0.6170 ± 0.026	0.6200 ± 0.023	0.6560 ± 0.050
	global	0.6437 ± 0.039	0.6000 ± 0.086	0.6809 ± 0.038	0.6737 ± 0.032	0.6824 ± 0.027
	local	0.6640 ± 0.019	0.6641 ± 0.014	0.6828 ± 0.033	0.6648 ± 0.020	0.7230 ± 0.032
	UMHL	0.7172 ± 0.015	0.7096 ± 0.014	0.7249 ± 0.020	0.7171 ± 0.015	0.7504 ± 0.015
ADHD	prior	0.5957 ± 0.018	0.4962 ± 0.021	0.6554 ± 0.034	0.5957 ± 0.015	0.5810 ± 0.009
	global	0.6113 ± 0.027	0.5206 ± 0.022	0.6780 ± 0.040	0.6311 ± 0.024	0.5869 ± 0.019
	local	0.6351 ± 0.033	0.5345 ± 0.012	0.7157 ± 0.050	0.6365 ± 0.026	0.5984 ± 0.007
	UMHL	0.6688 ± 0.017	0.5588 ± 0.020	0.7428 ± 0.028	0.6688 ± 0.017	0.6346 ± 0.021

## 5 Conclusion

In this work, we proposed the unified multi-view hypergraph learning framework, which integrates both knowledge-driven and data-driven approaches to model the high-order interactions within brain networks. By leveraging prior knowledge of functional brain subnetworks and adaptive data-driven techniques, our framework captures more nuanced and disease-specific associations compared to traditional graph and hypergraph methods. The proposed framework not only enhances the understanding of complex brain connectivity but also provides a robust and flexible tool for diagnosing neurodevelopmental disorders.

The proposed method also has some limitations. In actual clinical deployment, multi-branch models may indeed pose challenges related to high computational costs and deployment complexity. In addition, knowledge-driven high-order correlation patterns were only used with predefined eight functional brain networks, and task-related or disease-specific functional connectivity patterns have not yet been introduced. In our future work, we will introduce more modalities to mine different dimensions and types of higher-order correlation patterns.

## Data Availability

Publicly available datasets were analyzed in this study. This data can be found at: https://fcon_1000.projects.nitrc.org/indi/abide/; http://preprocessed-connectomes-project.org/adhd200/index.html.

## Footnotes

^1^ http://rfmri.org/DPARSF
